# Memory concerns in the early Alzheimer's disease prodrome: Regional association with tau deposition

**DOI:** 10.1016/j.dadm.2018.03.001

**Published:** 2018-03-24

**Authors:** Cecily G. Swinford, Shannon L. Risacher, Arnaud Charil, Adam J. Schwarz, Andrew J. Saykin

**Affiliations:** a Department of Radiology and Imaging Sciences, Center for Neuroimaging, Indiana University School of Medicine, Indianapolis, IN, USA; b Indiana Alzheimer Disease Center, Indiana University School of Medicine, Indianapolis, IN, USA; c Stark Neuroscience Research Institute, Indiana University School of Medicine, Indianapolis, IN, USA; d Eli Lilly and Company, Indianapolis, IN, USA; e Department of Psychological and Brain Science, Indiana University, Bloomington, IN, USA

**Keywords:** Significant memory concern, SMC, Subjective cognitive decline, SCD, Early mild cognitive impairment, EMCI, Neuroimaging, AV1451, Tau PET, Alzheimer's disease neuroimaging initiative, ADNI

## Abstract

**Introduction:**

Relationship between self– and informant memory concerns and tau aggregation was assessed in adults at risk for Alzheimer's disease (AD).

**Methods:**

Regional mean standardized uptake value ratios were extracted from [^18^F]flortaucipir positron emission tomography (PET) scans of 82 at-risk adults in the Alzheimer's Disease Neuroimaging Initiative (ADNI) cohort. Associations between self– and informant ECog memory scores and tau aggregation were analyzed on both regional and voxelwise bases. Analyses were completed both on the whole sample and restricted to amyloid-positive individuals only.

**Results:**

Memory concerns were associated with tau aggregation. Self-perception was more associated with frontal tau. In contrast, informant scores were more associated with parietal tau. This source-by-region interaction was more prominent in amyloid-positive participants and observed in both regional and voxelwise analyses.

**Discussion:**

Quantitative assessment of perceived memory functioning may be useful for screening older adults at risk for Alzheimer's disease. Individuals and their informants may provide complementary information relating to the anatomical distribution of tau.

## Highlights

• Both self– and informant memory concerns are associated with tau aggregation in at-risk adults.
• Self-concerns are more associated with frontal tau compared to a parietal pattern for informant concerns.
• This pattern is strongest in amyloid-positive participants.

## 1 Introduction

Alzheimer's disease (AD), the leading cause of neurodegenerative dementia associated with aging, affects over 5 million adults in the United States and is predicted to increase to 16 million affected by 2050 [1]. There are presently no approved pharmacological treatments that can stop the progression of AD. Treatment is likely to be most effective during the preclinical or early prodromal stages of AD, before substantial permanent neurodegenerative and cognitive damage has occurred. Therefore, there has been considerable recent interest in measures to identify older adults at highest risk for progression to AD who may benefit most from early intervention [2], [3].

Adults with subjective cognitive decline (SCD) in the presence of normal neuropsychological test scores are at an increased risk of progression to AD. These adults have been shown to progress to mild cognitive impairment (MCI) and eventually AD or a related dementia at a higher rate than cognitively normal (CN) adults who do not have SCD [2], [4], [5], [6], [7], [8]. Adults with SCD also show subtle, subclinical differences in objective cognitive performance compared to adults without SCD and experience more functional decline over time [9]. Therefore, it has been suggested that SCD is potentially a preclinical stage of AD [9]. However, SCD has also been linked to depression, other affective disorders, and personality traits [7], [10], [11], [12], [13]. Therefore, it is necessary to determine the factors that influence the clinical and prognostic significance of SCD.

In addition to capturing self-based estimates of SCD, investigators often also assess the extent of concerns about cognitive decline from an informant (spouse, child, other caregiver, or clinician). Informant-based cognitive concerns are particularly important in the later stages of cognitive decline, when individuals' insight into their own cognitive problems diminishes and informant perceptions of cognition are more accurate [12], [14], [15]. In CN adults, however, self- and informant perceptions of cognitive decline are both predictive of future progression to MCI or AD, and the use of both measures together is a better predictor than either measure alone [5]. This finding suggests that, in very early stages of disease, both at-risk adults and their informants can provide important information about subclinical cognitive decline. Thus, using both sources of concern together may provide complementary information regarding subtle pathological changes in adults in very early preclinical stages of AD.

Many adults with SCD exhibit structural and pathological changes that are typically associated with MCI or AD. For example, adults with SCD show patterns of neurodegeneration, such as decreased gray matter and hippocampal volumes, that are similar to those seen in adults with MCI [16], [17], [18]. Similarly, some adults with SCD and early mild cognitive impairment (EMCI) show AD-related pathology, such as amyloid plaques, tau tangles, and cerebrospinal fluid (CSF) profiles that are similar to those observed in AD (decreased levels of amyloid and increased levels of total and phosphorylated tau; [19], [20], [21]). Adults with SCD or EMCI who also show AD-like pathology are more likely to progress to later stages of MCI or AD [21], [22].

Tau aggregation is an important biomarker of disease severity along the spectrum of preclinical and clinical stages of AD. It has been previously established from measurements of tau in CSF and *postmortem* studies of brain tissue that tau aggregation correlates with both neurodegeneration and the resultant cognitive decline temporally and spatially during progression of AD [23], [24]. The recent development of tau-specific radiotracers has allowed *in vivo* positron emission tomography (PET) measurement and visualization of the spatial distribution of tau aggregation for the first time [25]. Tau radiotracers have permitted *in vivo* correlation of tau aggregation and other markers of disease progression, including increased cognitive decline, amyloid deposition, and CSF measures of amyloid and tau [26]. Spatial information about the tau anatomical distribution has also been shown to provide important clinical information; brain regions with high levels of tau aggregation often correspond to declines in cognitive functions related to those regions [27].

Because tau aggregation correlates spatially with brain areas implicated in cognitive decline, it is possible that self-based memory concerns correlate more strongly with tau aggregation in brain regions involved in introspection or internal thought processes, for example, the medial prefrontal cortex. More generally, the frontal cortex has been implicated in several aspects of conscious internal processing, such as planning, decision-making, and inhibition of actions by thinking through consequences. It is possible that preclinical pathological changes in frontal brain regions would be noticeable to the patient before causing outward changes in behavior due to impacts on the processes of internal thought. On the other hand, informant memory concerns may correlate more strongly with tau aggregation in brain regions typically seen in patients with MCI and AD, as these may be involved in common initial symptoms of AD (i.e., memory decline) that are more likely to be noticed by an observer.

To determine how self- and informant perceptions of cognitive decline are each related to tau deposition in the early stages of AD, we assessed the relationship between self- and informant scores on the memory subscale of the Test of Everyday Cognition (ECog; [28]), as well as the association of each with regional and global tau aggregation as measured by the tau PET radiotracer [^18^F]flortaucipir (T-807; AV-1451). Our goal was to evaluate the relationship between self– and informant memory concerns and tau deposition to investigate the biological basis for the predictive power of cognitive concerns and whether the self- and informant concerns could be utilized as part of a screening protocol to assess preclinical AD in individual adults. We included older adults enrolled in the Alzheimer's Disease Neuroimaging Initiative (ADNI) who were defined as CN controls, had significant memory concerns (SMCs), or had EMCI. These adults comprise a continuum of risk for developing clinical AD. A subset of the CN older adults are amyloid negative and apolipoprotein E gene (*APOE*) ε4 noncarriers and thus are at risk for AD due to age alone and are on the “low-risk” end of the continuum. On the “high-risk” end are adults with EMCI who have subtle cognitive decline, presence of self– and informant cognitive concerns, and are amyloid positive and/or *APOE* ε4 carriers. We examined the association of self– and informant ECog memory scores with one another and with tau aggregation in all participants. Following these analyses, we completed a subanalysis using only participants who are amyloid positive because these participants are at a relatively higher risk of developing AD. We hypothesized that self– and informant ECog memory scores would be mildly correlated with one another, and that higher ECog memory scores (indicating greater perceived memory decline) would be associated with increased levels of tau. Finally, we also hypothesized that self- and informant scores would potentially correlate with the distribution of tau aggregation in spatially different patterns of association throughout the brain.

## 2 Methods

### 2.1 Participants

Data used in the preparation of this article were obtained from the ADNI database (http://adni.loni.usc.edu; Supplementary Material). For up-to-date information, see http://www.adni-info.org. The 82 participants included in this study were diagnosed as CN older adults, SMC participants, or EMCI participants by the ADNI-2 procedures manual criteria (http://www.adni-info.org). According to these criteria, 40 CN participants had no subjective or informant complaint of cognitive decline and performed normally on the Wechsler Logical Memory Delayed Recall (LM-delayed) and the Mini–Mental State Examination (MMSE). 11 SMC participants expressed subjective memory concerns on the Cognitive Change Index (CCI; total score from first 12 items ≥ 16 [29], [30]) but had no significant informant complaint of cognitive decline and performed normally on the LM-delayed and MMSE. Finally, 31 EMCI participants had subjective, informant, and/or clinician complaint of cognitive decline, memory function approximately one standard deviation (SD) below normal on the LM-delayed, a MMSE total score greater than 24, and functioning at a level that precluded a diagnosis of AD. For the amyloid-positive group, we included only CN, SMC, and EMCI participants who were amyloid positive (n = 36; 15 CN, 4 SMC, and 17 EMCI) on the [^18^F]florbetapir PET scan closest to the [^18^F]flortaucipir PET scan, using data generated by the University of California, Berkeley, and downloaded from the ADNI site (global standardized uptake value ratio [SUVR] > 1.11 [31]). The percentage of subjects that were amyloid positive did not differ by the diagnostic group.

### 2.2 Clinical and cognitive assessments

Clinical and cognitive performance data were obtained from the ADNI database (http://adni.loni.usc.edu). Participants were given clinical and cognitive tests as described in the ADNI-2 manual (www.adni-info.org). For the primary analyses, we used self– and informant ECog memory scores, which are the averages of ratings on the eight questions in the memory section of the ECog. The ECog score from the test given closest in time to the [^18^F]flortaucipir scan was used [full sample: mean (SD) = 145.5 (186.8) days; amyloid-positive sample: mean (SD) = 128.4 (158.2) days], which did not differ by the diagnostic group.

### 2.3 [^18^F]flortaucipir PET scans

Preprocessed [^18^F]flortaucipir PET scans were downloaded from the ADNI Laboratory of Neuro Imaging (LONI; http://adni.loni.usc.edu) site. These scans were preprocessed using standard techniques in Statistical Parametric Mapping 8 (SPM8), including normalization to Montreal Neurologic Institute (MNI) space. Then, SUVR images were created by intensity normalization using a cerebellar crus reference region. Regional mean SUVR was extracted from subject-specific regions of interest (ROIs), including the bilateral mean parahippocamapal gyri, frontal lobe, parietal lobe, and global cortex. ROIs were generated from the closest in time structural MRI scan using FreeSurfer, version 5.1.

### 2.4 Statistical analysis

Partial Pearson correlations were used to assess the association between self– and informant ECog memory scores and with tau aggregation in the target ROIs using SPSS Statistics, version 24 (IBM Corporation, Somers, NY). Covariates for these analyses included age, sex, and years of education. Furthermore, *APOE* ε4 carrier status (where positive is having at least one *APOE* ε4 allele and negative is not having an *APOE* ε4 allele regardless of whether the other alleles are *APOE* ε2 or *APOE* ε3) and Geriatric Depression Scale (GDS) total score were tested as potential covariates in secondary analyses. The associations were assessed in the whole group of participants, as well as in amyloid-positive participants only and in amyloid-negative participants only.

χ^2^ tests were used to evaluate the association of sex, *APOE* ε4 positivity, or amyloid positivity with the diagnostic group. A one-way analysis of variance (ANOVA) was used to assess differences in age, years of education, a composite memory score, GDS score, self–ECog memory score, and informant ECog memory score by the diagnostic group. Again, these associations were examined using all participants and in amyloid-positive participants only. Post hoc differences were evaluated after Bonferroni adjustment for multiple comparisons, with *P* < .05 after correction considered significant.

### 2.5 Voxelwise analysis

In addition, the associations of self– and informant ECog memory scores and [^18^F]flortaucipir SUVR were evaluated on a whole-brain voxelwise basis in SPM8 using a multiple linear regression model, masked for the gray plus white matter, and including age, sex, and years of education as covariates. Significance was set at a voxelwise threshold of *P* < .005 (uncorrected for multiple comparisons) and a minimum cluster size (k) of 675 voxels, which corresponds to a clusterwise threshold of *P* < .05 (familywise error [FWE] correction for multiple comparisons). Talairach Daemon was used to identify brain regions of significant clusters. As in the regional analyses, the voxelwise analyses included an initial analysis using the whole sample and a follow-up analysis included amyloid-positive participants only.

## 3 Results

### 3.1 Demographics

Effects of diagnosis on demographics, neuropsychological test scores, and self– and informant ECog memory scores are shown in Table 1 for all participants and in Supplementary Table 1 for amyloid-positive participants. For all participants, as expected, participants with EMCI had lower memory performance with lower composite scores than CN participants (*P* = .007). Participants with EMCI had higher informant ECog memory scores than CN participants (*P* < .001). Sex was significantly different across diagnostic groups such that men made up a greater percentage of the EMCI group, whereas women made up a greater percentage of the CN group (*P* = .049). There were no significant differences in age, years of education, GDS scores, *APOE* ε4 positivity, amyloid positivity, or self–ECog memory scores between diagnostic groups. For amyloid-positive participants, participants with EMCI had lower memory composite scores than CN participants (*P* = .045). Participants with EMCI also had higher informant ECog memory scores than CN participants (*P* = .019). No significant differences in age, years of education, sex, GDS scores, *APOE* ε4 genotype, or self–ECog memory scores were observed between diagnostic groups.

**Table 1:** Demographic information for all participants

Variable	CN (n = 40)	SMC (n = 11)	EMCI (n = 31)	*P* value	Significant pair comparisons∗
Age (y)	76.48 (7.211)	71.55 (5.11)	75.32 (7.29)	.125	None
Education (y)	16.03 (2.37)	16.00 (2.49)	17.03 (2.39)	.184	None
Sex (M, F)	17, 23	5, 6	22, 9	.049	N/A
*APOE* ε4 positivity (%)	42.5	45.5	25.8	.256	N/A
Amyloid positivity (%)	37.5	36.4	54.8	.297	N/A
Memory composite	1.23 (0.67)	1.20 (0.63)	0.73 (0.69)	.007	EMCI < CN
GDS total	1.21 (1.59)	0.91 (0.83)	1.52 (1.88)	.527	None
Self–ECog memory	1.77 (0.66)	1.88 (0.68)	2.11 (0.68)	.114	None
Informant ECog memory	1.41 (0.48)	1.52 (0.50)	2.02 (0.77)	<.001	EMCI > CN

Abbreviations: *APOE*, apolipoprotein E; CN, cognitively normal; ECog, Test of Everyday Cognition; EMCI, early mild cognitive impairment; M, male; F, female; GDS, Geriatric Depression Scale; SMC, significant memory concern.

∗ *P* < .05 (Bonferroni adjustment for multiple comparisons).

### 3.2 Association of self– and informant ECog memory scores

Self–ECog memory scores were only mildly correlated with informant ECog memory scores, after covariate adjustment, when all participants were included (r = 0.362, r_p_ = 0.001). When only amyloid-positive participants were included, the correlation between self– and informant ECog memory scores did not reach statistical significance (r = 0.243, r_p_ = 0.173).

### 3.3 Regional analysis in all participants

**Fig. 1:**
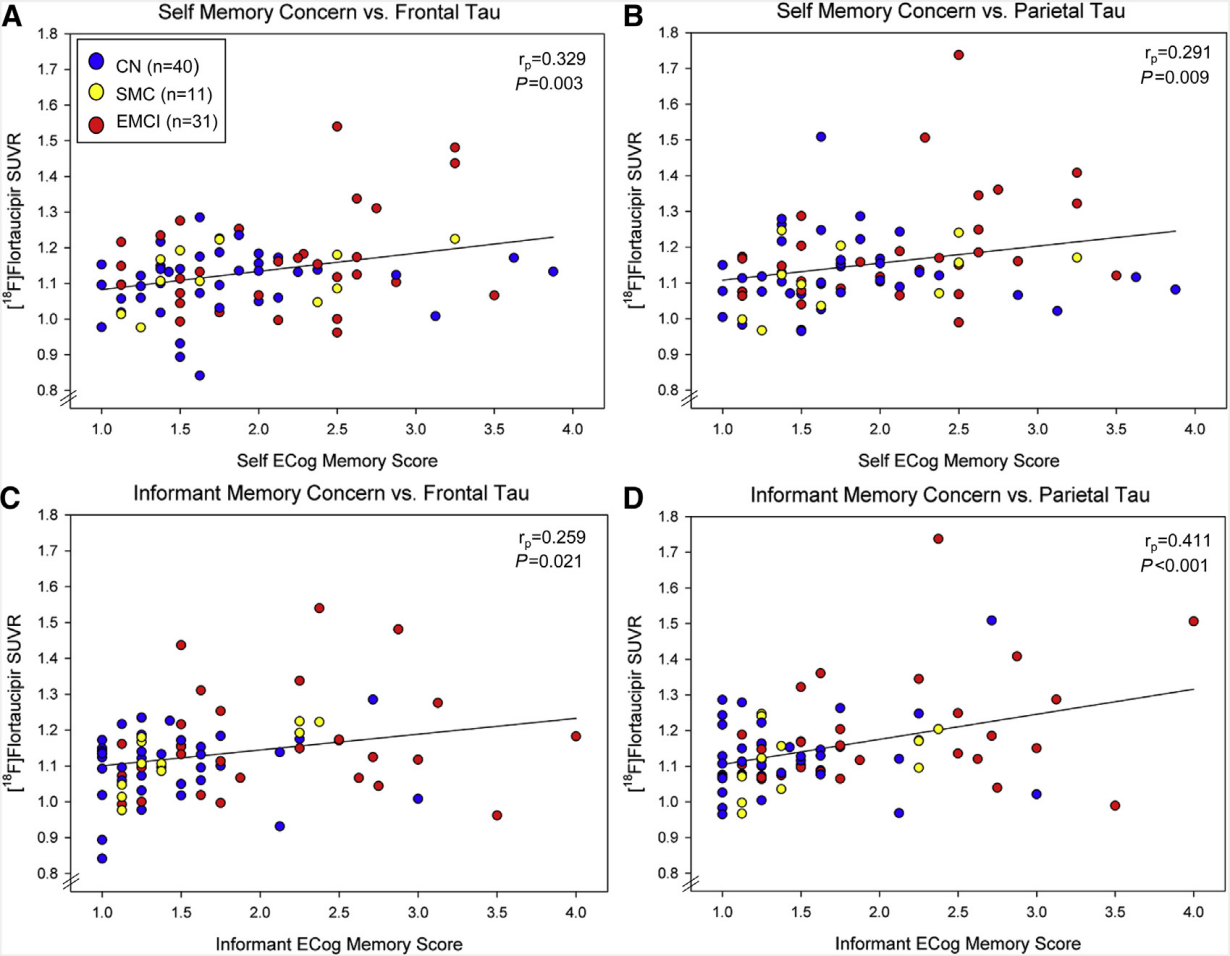
Association of self– and informant memory concerns with tau. Self–ECog memory scores associate more strongly with (A) tau aggregation in the frontal lobe (r_p_ = 0.329, *P* = .003) than (B) that in the parietal lobe (r_p_ = 0.291, *P* = .009). Alternatively, informant ECog memory scores associate more strongly with tau aggregation (D) in the parietal lobe (r_p_ = 0.411, *P* < .001) than (C) that in the frontal lobe (r_p_ = 0.259, *P* = .021). However, all associations are significant (*P* < .05). Scatterplots show raw data points; r_p_ values and *P* values were generated from a model with age, sex, and years of education as covariates. Abbreviations: CN, cognitively normal; ECog, Test of Everyday Cognition; EMCI, early mild cognitive impairment; SUVR, standardized uptake value ratio.

Self–ECog memory scores were significantly correlated with tau aggregation, after covariate adjustment, in all four ROIs (parahippocampal: r_p_ = 0.293, *P* = .009; frontal: r_p_ = 0.329, *P* = .003; parietal: r_p_ = 0.291, *P* = .009; and global: r_p_ = 0.306, *P* = .006; Fig. 1A). Informant ECog memory scores were similarly significantly associated with tau aggregation in all four regions, but the correlation with tau aggregation in the parietal region was the strongest (parahippocampal: r_p_ = 0.283, *P* = .011; frontal: r_p_ = 0.259, *P* = .021; parietal: r_p_ = 0.411, *P* < .001; and global: r_p_ = 0.296, *P* = .008; Fig. 1B).

### 3.4 Regional analysis in amyloid-positive participants

**Fig. 2:**
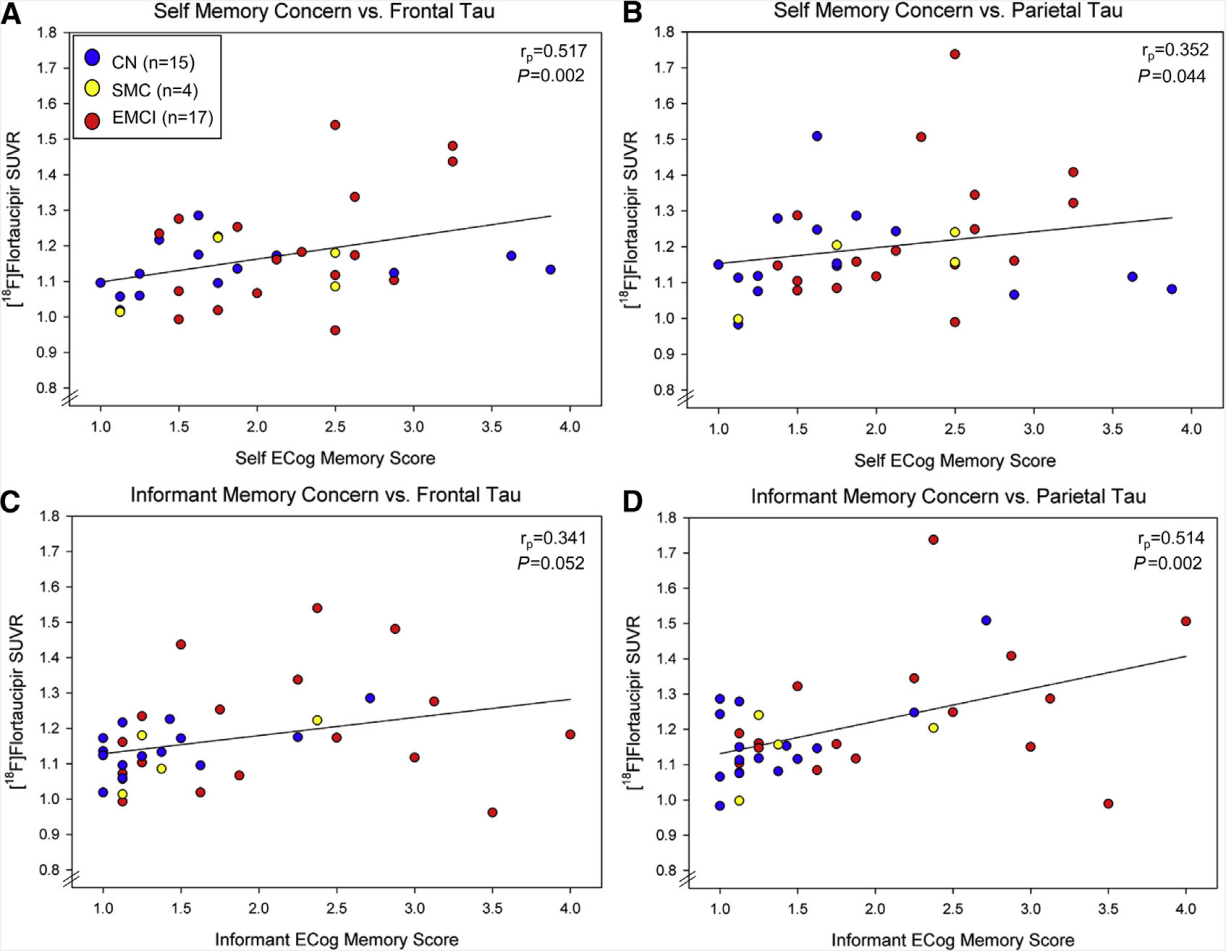
Association of self– and informant memory concerns with tau in amyloid-positive participants. Similar to the pattern observed in all participants, self–ECog memory scores associate more strongly with (A) tau aggregation in the frontal lobe (r_p_ = 0.517, *P* = .002) than (B) that in the parietal lobe (r_p_ = 0.352, *P* = .044). Contrary to this, informant ECog memory scores associate strongly with tau aggregation (D) in the parietal lobe (r_p_ = 0.514, *P* = .002) and (C) are borderline significantly associated with tau aggregation in the frontal lobe (r_p_ = 0.341, *P* = .052). Scatterplots show raw data points; r_p_ values and *P* values were generated from a model with age, sex, and years of education as covariates. Abbreviations: CN, cognitively normal; ECog, Test of Everyday Cognition; EMCI, early mild cognitive impairment; SUVR, standardized uptake value ratio.

When only amyloid-positive participants were included, self–ECog memory scores were again significantly correlated with tau aggregation in all four regions, and the strongest association was in the frontal lobe (parahippocampal: r_p_ = 0.411, *P* = .018; frontal: r_p_ = 0.517, *P* = .002; parietal: r_p_ = 0.352, *P* = .044; and global: r_p_ = 0.459, *P* = .007; Fig. 2A). Alternatively, informant ECog memory scores were significantly correlated with tau aggregation in all regions except for the frontal region (parahippocampal: r_p_ = 0.347, *P* = .048; frontal: r_p_ = 0.341, *P* = .052; parietal: r_p_ = 0.514, *P* = .002; and global: r_p_ = 0.416, *P* = .016), and the strongest correlation with tau aggregation was in the parietal region (Fig. 2B). When only amyloid-negative participants were included, neither self– nor informant ECog memory scores were correlated with tau aggregation in any of the ROIs (*data not shown*).

### 3.5 Interaction analysis

**Fig. 3:**
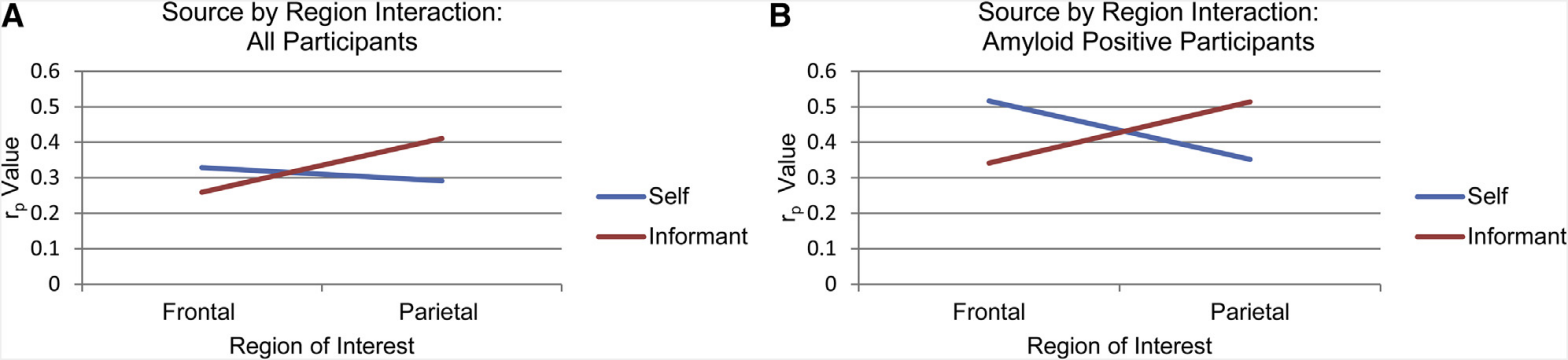
Source-by-region interaction analysis. Self– and informant ECog memory scores are most strongly associated with tau aggregation in frontal and parietal regions, respectively. The source-by-region interactions (A) for all participants (r_p_ = 0.283, *P* = .012) and (B) for amyloid positive participants (r_p_ = 0.47, *P* = .006) are statistically significant. Graphs plot r_p_ values from partial Pearson correlations between each ECog memory score and region of interest with age, sex, and years of education as covariates. Statistical significance of the source-by-region interactions was determined by a partial Pearson correlation between the source difference score (self-score minus informant score) and the region difference score (frontal SUVR minus parietal SUVR). Covariates were age, sex, and years of education. Abbreviations: SUVR, standardized uptake value ratio; ECog, Test of Everyday Cognition.

To determine whether there is a significant interaction of source of cognitive concern (self vs. informant) and location of tau deposition (frontal vs. parietal), we calculated the difference scores by subtracting informant ECog memory scores from self-ECog memory scores and mean frontal parietal from mean frontal [^18^F]flortaucipir SUVR. We then evaluated the association between these two difference scores. A statistically significant concern source-by-region interaction was observed in which self-scores are preferentially associated with tau aggregation in frontal regions and informant scores are preferentially associated with tau aggregation in parietal regions, both in all participants and in amyloid-positive participants (Fig. 3).

Including either *APOE* ε4 allele positivity or GDS scores as additional covariates did not change the observed pattern of results in either the full sample or the amyloid-positive only sample (*data not shown*).

### 3.6 Voxelwise analyses

**Fig. 4:**
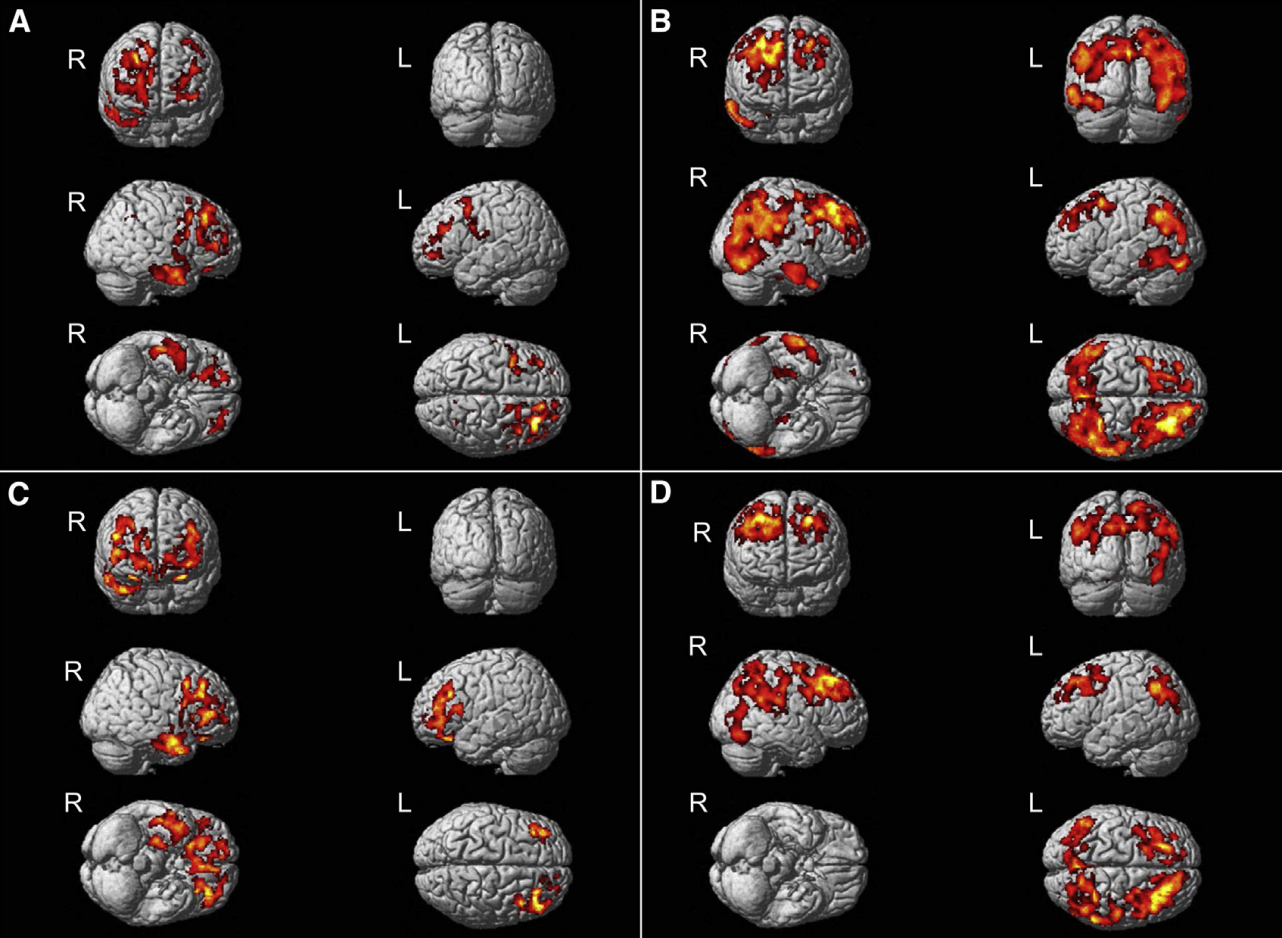
Voxelwise association between self– and informant memory concerns and tau aggregation. (A) Voxelwise analysis of [^18^F]flortaucipir PET scans shows statistically significant clusters of association between self–ECog memory scores and tau aggregation across all participants, in primarily bilateral frontal and right temporal lobe regions. (B) Informant ECog memory scores were associated with tau deposition on [^18^F]flortaucipir PET in bilateral frontal, bilateral parietal, bilateral temporal, and bilateral occipital lobes, as well as the bilateral cerebellum across all participants. In amyloid-positive participants only, voxelwise analysis showed statistically significant clusters of association between (C) self–ECog memory scores and tau aggregation correlation in bilateral frontal lobes, right temporal lobe, and right cerebellum and between (D) informant ECog memory scores and tau aggregation correlation in the left parietal, bilateral occipital, and bilateral frontal lobes. The figures are displayed at a voxelwise threshold of *P* < .005 (uncorrected for multiple comparisons); minimum cluster size (k) = 675 voxels. Abbreviations: PET, positron emission tomography; ECog, Test of Everyday Cognition.

To assess the spatial differences between the associations with tau and self- and informant ECog memory scores without the bias of using our predetermined ROIs, we performed voxelwise analyses in SPM8. In all participants, the self–ECog memory scores were associated with tau in the following regions: bilateral frontal lobe (middle, medial, superior, and inferior frontal gyri), left frontal subgyral region, right temporal lobe (middle, superior, inferior, and fusiform gyri and subgyral region), bilateral precentral gyrus, left postcentral gyrus, bilateral cingulate gyrus, bilateral anterior cingulate, bilateral posterior cingulate, right parahippocampal gyrus, right precuneus, right insula, right uncus, and anterior lobe of right cerebellum (clusterwise threshold of *P* < .05 FWE; Supplementary Table 2; Fig. 4A). Informant ECog memory scores were associated with tau aggregation in the following regions: bilateral frontal lobe (middle, medial, and superior frontal gyri), right frontal subgyral region, bilateral temporal lobe (middle, inferior, and fusiform gyri), left superior temporal gyrus, right supramarginal gyrus, bilateral precentral gyrus, right inferior parietal lobule, right occipital lobe (middle and superior occipital gyri), left inferior occipital gyrus, bilateral precuneus, left cuneus, bilateral posterior cingulate, bilateral cingulate gyrus, right insula, bilateral posterior lobe of cerebellum, and anterior lobe of left cerebellum (clusterwise threshold of *P* < .05 FWE; Supplementary Table 3; Fig. 4B).

In amyloid-positive participants, self–ECog memory scores were associated with tau aggregation in the following regions: bilateral frontal lobe (middle, inferior, and subcallosal gyri), left medial frontal gyrus, right temporal lobe (middle and inferior temporal gyri), right precentral gyrus, bilateral anterior cingulate, right putamen, right uncus, right insula, and anterior lobe of right cerebellum (clusterwise threshold of *P* < .05 FWE; Supplementary Table 4; Fig. 4C). Informant ECog memory scores were associated with tau aggregation in the following regions: bilateral frontal lobe (middle, medial, superior, and inferior gyri and subgyral region), bilateral middle temporal gyrus, right supramarginal gyrus, left superior temporal gyrus, bilateral precentral gyrus, right postcentral gyrus, bilateral precuneus, bilateral cingulate gyrus, bilateral posterior cingulate, left cuneus, right insula, and posterior lobe of right cerebellum (clusterwise threshold of *P* < .05 FWE; Supplementary Table 5; Fig. 4D).

## 4 Discussion

In this study, we found that self– and informant memory concerns are both correlated with tau aggregation in at-risk adults, but that the overall spatial patterns of associations differ between the two measures of subjective memory concern. We found that self– and informant ECog memory scores are only mildly correlated with one another in at-risk adults and are not correlated with one another in the subset of amyloid-positive adults, suggesting that the two measures of subjective memory complaints may be somewhat independent and provide complementary information. The results from our ROI-based analyses suggest that self-based memory complaints are most strongly associated with tau aggregation in the frontal lobes, whereas informant memory complaints are most strongly associated with tau aggregation in the parietal lobes. These differences in patterns of correlation were enhanced when only amyloid-positive participants were included, and significant source-by-region interactions were found in both the full analysis and the subanalysis of amyloid-positive individuals. There were no correlations found when only amyloid-negative participants were included, suggesting that the patterns of association found are specifically present in adults at higher risk for developing AD, which supports the idea that these subjective memory tools may be useful for optimizing screening techniques in the population of at-risk adults. In addition to our regional analyses, we evaluated the associations with voxelwise analyses to lessen the bias imposed by our predetermined ROIs. Although these analyses revealed regions of overlap between the correlations of tau aggregation with self– and informant memory concerns, they resulted in the same general patterns found with the ROI-based analyses. Specifically, both self– and informant memory concerns were associated with tau aggregation in the frontal and temporal lobes when all participants were included, while the informant memory concerns were also associated with tau aggregation in posterior brain regions, including the parietal and occipital lobes. When only amyloid-positive participants were included, the self-based memory concerns were significantly associated with tau aggregation in bilateral frontal lobes and the right temporal lobe, while the informant memory concerns were also correlated with tau burden in the left parietal lobe and bilateral occipital lobes.

Our data suggest that self-based memory concerns correlate more strongly with tau aggregation in regions typical of conscious internal thought processes (i.e., frontal lobe, specifically the medial prefrontal region), while informant memory concerns correlate more strongly with tau aggregation in posterior regions typical of more progressed MCI and AD patients. These posterior regions may be involved with the more outward signs of cognitive decline that can be noticed by an observer who knows the individual well. In support of this implication of our results, the voxelwise analysis revealed that only self-based memory concerns were correlated with tau aggregation in the bilateral anterior cingulate, while only informant memory concerns were correlated with tau aggregation in the supramarginal gyri. The anterior cingulate has been shown to be involved in decision-making [32], while the supramarginal gyrus is involved in language perception and processing [33]. While correlations within these specific brain regions are interesting, future research focused on the relationship between subjective memory concerns and regional pathology will be necessary to confirm the relationships that we observe here.

Because both self-based cognitive complaints and pathological changes in the frontal lobe have been shown to be correlated with depression [7], [10], [11], [34], we used GDS scores as a covariate along with age, sex, and years of education to ensure that depressive symptomology was not a confound in the observed correlations. The inclusion of this score as a covariate did not significantly change the patterns of association; the self-based memory concerns were still significantly correlated to tau in the frontal region (*data not shown*). Individuals' depressive symptoms have been shown to increase informant-based cognitive complaints in other studies as well [15], [35], but the pattern of association between informant based memory concerns and tau was not changed when GDS scores were used as a covariate (*data not shown*). It is still possible that some of the participants experienced subtle depressive symptoms that were not reflected in the GDS scores but still influenced their perceptions of their own cognitive functioning. Future studies exploring the interactions between depression, cognitive concerns, and AD pathophysiology are warranted.

Overall, our findings suggest that subjective memory concerns have the potential to be optimized and used as part of a screening protocol for AD-related pathology and disease progression in adults with preclinical or prodromal stages of AD. Along with the differing regions of tau association between the two measures of subjective memory concern, this finding suggests that using both self- and informant measures together may provide important complementary information, such that high scores on both measures may suggest a greater overall tau burden in the brain. Finally, these results provide a potential biological explanation for the previous finding that using self– and informant memory concerns together is a better predictor of future progression to MCI or AD than either measure alone [5].

This study does have some potential limitations. First, our sample size, especially in the amyloid-positive subanalysis, was relatively small, which could lead to bias. Although we included all available CN, SMC, and EMCI participants in ADNI-2 who had tau scans at the time of our analyses, we recognize that the CN group had more *APOE* ε4 carriers than expected in the general population and that the amyloid positivity surprisingly did not differ between the diagnostic groups. Second, we also acknowledge that there may have been unforeseen selection bias in the ADNI-2 study. Future studies in larger samples will help to support the present findings. In addition, the SMC group from ADNI-2 was defined on the basis of subjective- or self-based memory complaints but not informant-based concerns. Therefore, there was not a group in this study for participants who had solely informant-based complaints in the context of normal or above average cognitive functioning. Third, this study only evaluated cross-sectional data; future studies with longitudinal data are needed to address changes in self- and informant concerns across the disease spectrum and their association with changes in AD pathophysiology. Evaluating whether CN individuals who later progress to AD show different patterns of self– and/or informant cognitive concerns and differing patterns of association of these concerns with AD pathology could further validate quantitative assessment of memory concerns as a useful screening tool. Finally, we used Talairach Daemon to define the brain regions from our voxelwise analysis. Although this atlas is not specific to our study or to an aging population, we felt that it was the most appropriate tool to use in this case. A standardized atlas specific for older adults has not been defined in the literature, and our small sample size kept us from producing our own study-specific atlas. We do not expect that extensive atrophy should confound the results of the Talairach Daemon atlas in our preclinical AD population, and we visually inspected labeled regions for accuracy. However, we acknowledge that use of this nonspecific atlas may have caused minor labeling issues in our aging participants.

In summary, we demonstrated that both self– and informant memory concerns are associated with tau aggregation in adults at risk for AD. Furthermore, we found that self– and informant memory concerns correlate with tau aggregation in spatially different patterns of association throughout the brain. Overall, these findings suggest that both self– and informant memory concerns have the potential to be used as part of a screening process for preclinical AD and that they may also provide complementary information about both future conversion and AD-related pathological patterns. Although future studies are needed, it may be the case that the discrepancy between self– and informant memory concerns could help determine the location of tau aggregation for individuals, thus adding information for better detection, diagnosis, and prognosis of future decline. Future studies with larger sample sizes and longitudinal data will help to further elucidate which measures of perceived cognitive decline and which patterns of tau aggregation are most accurate at predicting progression to AD.

**Research in Context**

1. Systematic review: To review what is already known about relationships between self– and informant cognitive concerns and Alzheimer's disease (AD) biomarkers, we searched PubMed for: “cognitive complaints (CC),” “neuroimaging,” and “Alzheimer's disease (AD).” We then summarized the findings from the resulting articles into a brief overview of the relationships between self– and informant cognitive concerns and AD biomarkers.
2. Interpretation: Our results suggest that both self– and informant memory concerns have the potential to be used as part of a screening process for preclinical AD and that self- and informant concerns will likely provide complementary information about AD-related pathological patterns and future conversion.
3. Future directions: Studies with larger sample sizes and longitudinal data will help to further elucidate the self- and informant concerns and tau aggregation patterns that most accurately predict progression to AD.
