# Supplementary material for: Memory concerns in the early Alzheimer's disease prodrome: Regional association with tau deposition
Source: Alzheimers Dement (Amst). 2018 Mar 24;10:322–31. doi: 10.1016/j.dadm.2018.03.001 (PMC5956937; doi:10.1016/j.dadm.2018.03.001)
Supplement: Supplementary Material — Material [file mmc1.docx]

**Supplementary Material**

*Alzheimer’s Disease Neuroimaging Initiative (ADNI)*

Data used in the preparation of this article were obtained from ADNI (<http://adni.loni.usc.edu>). ADNI was launched in 2003 by the National Institute on Aging (NIA), the National Institute of Biomedical Imaging and Bioengineering (NIBIB), the Food and Drug Administration (FDA), private pharmaceutical companies and non-profit organizations, as a $60 million, 5-year public-private partnership. The primary goal of ADNI has been to test whether serial magnetic resonance imaging (MRI), positron emission tomography (PET), other biological markers, and clinical and neuropsychological assessment can be combined to measure the progression of mild cognitive impairment (MCI) and early Alzheimer’s disease (AD). Determination of sensitive and specific markers of very early AD progression is intended to aid researchers and clinicians to develop new treatments and monitor their effectiveness, as well as lessen the time and cost of clinical trials.

The Principal Investigator of this initiative is Michael W. Weiner, MD, VA Medical Center and University of California-San Francisco. ADNI is the result of efforts of many co-investigators from a broad range of academic institutions and private corporations, and subjects have been recruited from over 50 sites across the U.S. and Canada. The initial goal of ADNI was to recruit 800 subjects but ADNI has been followed by ADNI-GO and ADNI-2. To date these three protocols have recruited over 1500 adults, ages 55 to 90, to participate in the research, consisting of cognitively normal older individuals, people with early or late MCI, and people with early AD. The follow up duration of each group is specified in the protocols for ADNI-1, ADNI-2 and ADNI-GO. Subjects originally recruited for ADNI-1 and ADNI-GO had the option to be followed in ADNI-2. Further information can be found at <http://www.adni-info.org/> and in previous reports [^1-18^](#_ENREF_1). Informed consent was obtained according to the Declaration of Helsinki.

References

1. Jack CR, Jr., Bernstein MA, Borowski BJ, et al. Update on the magnetic resonance imaging core of the Alzheimer's disease neuroimaging initiative. Alzheimer's & dementia : the journal of the Alzheimer's Association 2010;6:212-220.

2. Jagust WJ, Bandy D, Chen K, et al. The Alzheimer's Disease Neuroimaging Initiative positron emission tomography core. Alzheimer's & dementia : the journal of the Alzheimer's Association 2010;6:221-229.

3. Saykin AJ, Shen L, Foroud TM, et al. Alzheimer's Disease Neuroimaging Initiative biomarkers as quantitative phenotypes: Genetics core aims, progress, and plans. Alzheimer's & dementia : the journal of the Alzheimer's Association 2010;6:265-273.

4. Trojanowski JQ, Vandeerstichele H, Korecka M, et al. Update on the biomarker core of the Alzheimer's Disease Neuroimaging Initiative subjects. Alzheimer's & dementia : the journal of the Alzheimer's Association 2010;6:230-238.

5. Weiner MW, Aisen PS, Jack CR, Jr., et al. The Alzheimer's disease neuroimaging initiative: progress report and future plans. Alzheimer's & dementia : the journal of the Alzheimer's Association 2010;6:202-211 e207.

6. Aisen PS, Petersen RC, Donohue M, Weiner MW, Alzheimer's Disease Neuroimaging I. Alzheimer's Disease Neuroimaging Initiative 2 Clinical Core: Progress and plans. Alzheimer's & dementia : the journal of the Alzheimer's Association 2015;11:734-739.

7. Beckett LA, Donohue MC, Wang C, et al. The Alzheimer's Disease Neuroimaging Initiative phase 2: Increasing the length, breadth, and depth of our understanding. Alzheimer's & dementia : the journal of the Alzheimer's Association 2015;11:823-831.

8. Franklin EE, Perrin RJ, Vincent B, et al. Brain collection, standardized neuropathologic assessment, and comorbidity in Alzheimer's Disease Neuroimaging Initiative 2 participants. Alzheimer's & dementia : the journal of the Alzheimer's Association 2015;11:815-822.

9. Hendrix JA, Finger B, Weiner MW, et al. The Worldwide Alzheimer's Disease Neuroimaging Initiative: An update. Alzheimer's & dementia : the journal of the Alzheimer's Association 2015;11:850-859.

10. Jack CR, Jr., Barnes J, Bernstein MA, et al. Magnetic resonance imaging in Alzheimer's Disease Neuroimaging Initiative 2. Alzheimer's & dementia : the journal of the Alzheimer's Association 2015;11:740-756.

11. Jagust WJ, Landau SM, Koeppe RA, et al. The Alzheimer's Disease Neuroimaging Initiative 2 PET Core: 2015. Alzheimer's & dementia : the journal of the Alzheimer's Association 2015;11:757-771.

12. Jones-Davis DM, Buckholtz N. The impact of the Alzheimer's Disease Neuroimaging Initiative 2: What role do public-private partnerships have in pushing the boundaries of clinical and basic science research on Alzheimer's disease? Alzheimer's & dementia : the journal of the Alzheimer's Association 2015;11:860-864.

13. Kang JH, Korecka M, Figurski MJ, et al. The Alzheimer's Disease Neuroimaging Initiative 2 Biomarker Core: A review of progress and plans. Alzheimer's & dementia : the journal of the Alzheimer's Association 2015;11:772-791.

14. Liu E, Luthman J, Cedarbaum JM, et al. Perspective: The Alzheimer's Disease Neuroimaging Initiative and the role and contributions of the Private Partner Scientific Board (PPSB). Alzheimer's & dementia : the journal of the Alzheimer's Association 2015;11:840-849.

15. Saykin AJ, Shen L, Yao X, et al. Genetic studies of quantitative MCI and AD phenotypes in ADNI: Progress, opportunities, and plans. Alzheimer's & dementia : the journal of the Alzheimer's Association 2015;11:792-814.

16. Toga AW, Crawford KL. The Alzheimer's Disease Neuroimaging Initiative informatics core: A decade in review. Alzheimer's & dementia : the journal of the Alzheimer's Association 2015;11:832-839.

17. Weiner MW, Veitch DP, Aisen PS, et al. Impact of the Alzheimer's Disease Neuroimaging Initiative, 2004 to 2014. Alzheimer's & dementia : the journal of the Alzheimer's Association 2015;11:865-884.

18. Weiner MW, Veitch DP, Aisen PS, et al. 2014 Update of the Alzheimer's Disease Neuroimaging Initiative: A review of papers published since its inception. Alzheimer's & dementia : the journal of the Alzheimer's Association 2015;11:e1-120.

Supplementary Table 1

|  | CN (n=15) | SMC (n=4) | EMCI (n=17) | p-value | Significant Pair Comparisons* |
| --- | --- | --- | --- | --- | --- |
| Age (years) | 75.93 (5.56) | 71.25 (7.18) | 75.71 (8.19) | 0.485 | None |
| Education (years) | 16.67 (1.54) | 16.50 (1.73) | 16.18 (2.68) | 0.816 | None |
| Sex (M,F) | 5, 10 | 1, 3 | 12, 5 | 0.062 | N/A |
| *APOE* ε4 positivity (%) | 46.7% | 100% | 41.2% | 0.1 | N/A |
| Memory Composite | 1.39 (0.49) | 1.02 (0.91) | 0.75 (0.81) | 0.05 | EMCI<CN |
| GDS total | 0.93 (1.10) | 1.00 (0.82) | 1.18 (1.94) | 0.904 | None |
| Self ECog Memory | 1.88 (0.89) | 1.97 (0.66) | 2.24 (0.60) | 0.403 | None |
| Informant ECog Memory | 1.38 (0.50) | 1.53 (0.57) | 2.13 (0.91) | 0.02 | EMCI>CN |
| *APOE* = apolipoprotein E; CN = cognitively normal; ECog = Test of Everyday Cognition; EMCI = early mild cognitive impairment; M=male; F = female; GDS = Geriatric Depression Scale; SMC = significant memory concerns  * p<0.05 (Bonferroni adjustment for multiple comparisons) | | | | | |

Supplementary Table 2

| FWE p | Cluster Size (k) | T-value | Z-value | Cluster p (unc.) | Voxel p (unc.) | MNI Coordinates | | | Nearest Gray Matter Region |
| --- | --- | --- | --- | --- | --- | --- | --- | --- | --- |
|  |  |  |  |  |  | X | Y | Z |  |
| 0.017 | 844 | 4.36 | 4.11 | 0.001 | <0.001 | -26 | 46 | 14 | Left Medial Frontal Gyrus (BA 9) |
|  |  | 3.92 | 3.73 | 0.001 | <0.001 | -36 | 38 | 28 |  |
|  |  | 2.96 | 2.87 | 0.001 | 0.002 | -12 | 54 | 6 | Left Medial Frontal Gyrus (BA 10) |
|  |  | 2.69 | 2.62 | 0.001 | 0.004 | -18 | 52 | 14 |  |
|  |  | 3.67 | 3.52 | 0.001 | <0.001 | -30 | 50 | -4 | Left Frontal Sub-gyral |
|  |  | 3.32 | 3.2 | 0.001 | 0.001 | -38 | 50 | -4 |  |
|  |  | 3.41 | 3.28 | 0.001 | 0.001 | -20 | 56 | -2 | Left Superior Frontal Gyrus (BA 10) |
|  |  | 2.86 | 2.78 | 0.001 | 0.003 | -32 | 40 | -8 | Left Middle Frontal Gyrus (BA 47) |
|  |  | 3.34 | 3.21 | 0.001 | 0.001 | -38 | 26 | 36 | Left Middle Frontal Gyrus (BA 9) |
|  |  | 3.29 | 3.17 | 0.001 | 0.001 | -42 | 30 | 28 |  |
|  |  | 3.15 | 3.05 | 0.001 | 0.001 | -30 | 40 | 20 | Left Middle Frontal Gyrus (BA 10) |
|  |  | 3.14 | 3.04 | 0.001 | 0.001 | -42 | 40 | 0 | Left Inferior Frontal Gyrus (BA 47) |
|  |  | 2.83 | 2.75 | 0.001 | 0.003 | -44 | 36 | -8 |  |
|  |  | 2.83 | 2.75 | 0.001 | 0.003 | -16 | 50 | -10 | Left Anterior Cingulate (BA 32) |
| <0.001 | 5889 | 4.02 | 3.82 | <0.001 | <0.001 | -2 | -24 | 36 | Left Cingulate Gyrus (BA 23) |
|  |  | 3.45 | 3.32 | <0.001 | <0.001 | -4 | -42 | 26 | Left Posterior Cingulate (BA 30) |
|  |  | 3.47 | 3.33 | <0.001 | <0.001 | 42 | 44 | 8 | Right Middle Frontal Gyrus (BA 10) |
|  |  | 3.44 | 3.3 | <0.001 | <0.001 | 52 | 26 | 24 | Right Middle Frontal Gyrus (BA 46) |
|  |  | 4.12 | 3.91 | <0.001 | <0.001 | 26 | 28 | 38 | Right Middle Frontal Gyrus (BA 8) |
|  |  | 3.96 | 3.77 | <0.001 | <0.001 | 28 | 36 | 40 |  |
|  |  | 3.93 | 3.74 | <0.001 | <0.001 | 30 | 38 | -20 | Right Middle Frontal Gyrus (BA 11) |
|  |  | 3.8 | 3.63 | <0.001 | <0.001 | 40 | 32 | 38 | Right Middle Frontal Gyrus (BA 9) |
|  |  | 3.85 | 3.67 | <0.001 | <0.001 | 28 | 32 | 32 | Right Medial Frontal Gyrus (BA 9) |
|  |  | 3.74 | 3.58 | <0.001 | <0.001 | 12 | 54 | 24 |  |
|  |  | 3.69 | 3.53 | <0.001 | <0.001 | 16 | 42 | 32 |  |
|  |  | 3.68 | 3.52 | <0.001 | <0.001 | 14 | 14 | 50 | Right Medial Frontal Gyrus (BA 32) |
|  |  | 3.8 | 3.63 | <0.001 | <0.001 | 14 | 36 | 50 | Right Superior Frontal Gyrus (BA 8) |
|  |  | 3.64 | 3.48 | <0.001 | <0.001 | 24 | 52 | 14 | Right Superior Frontal Gyrus (BA 10) |
|  |  | 3.73 | 3.57 | <0.001 | <0.001 | 20 | 28 | -20 | Right Inferior Frontal Gyrus (BA 47) |
|  |  | 3.57 | 3.42 | <0.001 | <0.001 | 58 | 0 | 14 | Right Precentral Gyrus (BA 6) |
|  |  | 3.67 | 3.51 | <0.001 | <0.001 | 16 | -38 | 54 | Right Precuneus (BA 7) |
|  |  | 3.7 | 3.53 | <0.001 | <0.001 | 14 | -58 | 44 |  |
|  |  | 3.67 | 3.51 | <0.001 | <0.001 | 16 | -38 | 54 |  |
|  |  | 3.8 | 3.62 | <0.001 | <0.001 | 14 | 4 | 58 | Right Cingulate Gyrus (BA 24) |
|  |  | 4.04 | 3.83 | <0.001 | <0.001 | 4 | 2 | 32 |  |
|  |  | 3.66 | 3.5 | <0.001 | <0.001 | 10 | -10 | 48 |  |
|  |  | 3.68 | 3.52 | <0.001 | <0.001 | 10 | 14 | 44 | Right Cingulate Gyrus (BA 32) |
|  |  | 3.57 | 3.42 | <0.001 | <0.001 | 8 | 20 | 42 |  |
|  |  | 3.44 | 3.3 | <0.001 | <0.001 | 10 | 36 | 28 |  |
|  |  | 3.43 | 3.29 | <0.001 | <0.001 | 8 | -34 | 40 | Right Cingulate Gyrus (BA 31) |
|  |  | 3.59 | 3.44 | <0.001 | <0.001 | 12 | -42 | 54 |  |
|  |  | 3.46 | 3.32 | <0.001 | <0.001 | 6 | -46 | 24 | Right Posterior Cingulate (BA 30) |
|  |  | 3.45 | 3.32 | <0.001 | <0.001 | 22 | 52 | -2 | Right Anterior Cingulate (BA 10) |
|  |  | 3.56 | 3.41 | <0.001 | <0.001 | 50 | 12 | 18 | Right Insula (BA 13) |
|  |  | 3.47 | 3.33 | <0.001 | <0.001 | 44 | 10 | 22 |  |
|  |  | 3.46 | 3.32 | <0.001 | <0.001 | 6 | -46 | 24 | Right Posterior Cingulate (BA 30) |
|  |  | 3.45 | 3.32 | <0.001 | <0.001 | 22 | 52 | -2 | Right Anterior Cingulate (BA 10) |
|  |  | 3.65 | 3.5 | <0.001 | <0.001 | 10 | -50 | 8 | Right Cerebellum Anterior Lobe |
| 0.002 | 1288 | 4.29 | 4.05 | <0.001 | <0.001 | 54 | -2 | -24 | Right Fusiform Gyrus (BA 20) |
|  |  | 3.77 | 3.6 | <0.001 | <0.001 | 60 | -12 | -18 | Right Middle Temporal Gyrus (BA 21) |
|  |  | 3.64 | 3.49 | <0.001 | <0.001 | 48 | 4 | -32 |  |
|  |  | 3.4 | 3.27 | <0.001 | 0.001 | 56 | 6 | -6 | Right Superior Temporal Gyrus (BA 22) |
|  |  | 3.37 | 3.24 | <0.001 | 0.001 | 58 | 0 | -6 | Right Superior Temporal Gyrus (BA 38) |
|  |  | 3.32 | 3.2 | <0.001 | 0.001 | 60 | -2 | 0 | Right Superior Temporal Gyrus (BA 22) |
|  |  | 3.2 | 3.1 | <0.001 | 0.001 | 52 | 12 | -16 | Right Superior Temporal Gyrus (BA 38) |
|  |  | 3.85 | 3.67 | <0.001 | <0.001 | 50 | -20 | -28 | Right Inferior Temporal Gyrus (BA 20) |
|  |  | 3.49 | 3.35 | <0.001 | <0.001 | 46 | -8 | -34 |  |
|  |  | 3.71 | 3.55 | <0.001 | <0.001 | 46 | -26 | -24 | Right Parahippocampal Gyrus (BA 36) |
|  |  | 3.59 | 3.45 | <0.001 | <0.001 | 28 | 2 | -30 | Right Uncus (BA 28) |
|  |  | 3.58 | 3.43 | <0.001 | <0.001 | 40 | 2 | -34 | Right Uncus (BA 36) |
|  |  | 3.18 | 3.07 | <0.001 | 0.001 | 52 | -14 | -20 | Right Temporal Sub-gyral (BA 20) |
|  |  | 3.87 | 3.69 | 0.001 | <0.001 | -34 | 0 | 32 | No Gray Matter Found |
| 0.029 | 756 | 3.8 | 3.63 | 0.001 | <0.001 | -36 | 6 | 50 | Left Middle Frontal Gyrus (BA 6) |
|  |  | 3.6 | 3.45 | 0.001 | <0.001 | -24 | 0 | 46 |  |
|  |  | 3.49 | 3.35 | 0.001 | <0.001 | -46 | 8 | 46 |  |
|  |  | 3.01 | 2.92 | 0.001 | 0.002 | -34 | 8 | 60 |  |
|  |  | 3.01 | 2.91 | 0.001 | 0.002 | -28 | 14 | 60 |  |
|  |  | 3.07 | 2.97 | 0.001 | 0.001 | -28 | 14 | 40 | Left Middle Frontal Gyrus (BA 8) |
|  |  | 2.74 | 2.67 | 0.001 | 0.004 | -30 | 22 | 56 |  |
|  |  | 3.01 | 2.92 | 0.001 | 0.002 | -48 | 2 | 28 | Left Inferior Frontal Gyrus (BA 9) |
|  |  | 3.35 | 3.22 | 0.001 | 0.001 | -56 | 2 | 26 | Left Precentral Gyrus (BA 6) |
|  |  | 3.23 | 3.11 | 0.001 | 0.001 | -56 | -2 | 20 |  |
|  |  | 2.92 | 2.84 | 0.001 | 0.002 | -50 | 6 | 32 |  |
|  |  | 3.1 | 3 | 0.001 | 0.001 | -40 | 8 | 38 | Left Precentral Gyrus (BA 9) |
|  |  | 3.32 | 3.2 | 0.001 | 0.001 | -60 | -14 | 22 | Left Postcentral Gyrus (BA 43) |
|  |  | 3.53 | 3.39 | 0.001 | <0.001 | -22 | 10 | 44 | Left Cingulate Gyrus (BA 32) |
|  |  | 2.92 | 2.84 | 0.001 | 0.002 | -30 | -6 | 42 | No Gray Matter Found |

Supplementary Table 3

| FWE p | Cluster Size (k) | T-value | Z-value | Cluster p (unc.) | Voxel p (unc.) | MNI Coordinates | | | Nearest Gray Matter Region |
| --- | --- | --- | --- | --- | --- | --- | --- | --- | --- |
|  |  |  |  |  |  | X | Y | Z |  |
| <0.001 | 16758 | 4.56 | 4.28 | <0.001 | <0.001 | -44 | -70 | 32 | Left Middle Temporal Gyrus (BA 39) |
|  |  | 4.56 | 4.28 | <0.001 | <0.001 | -44 | -70 | 32 |  |
|  |  | 4.24 | 4.01 | <0.001 | <0.001 | -52 | -62 | 22 | Left Middle Temporal Gyrus (BA 19) |
|  |  | 4.74 | 4.43 | <0.001 | <0.001 | -48 | -56 | 40 | Left Superior Temporal Gyrus (BA 39) |
|  |  | 4.81 | 4.49 | <0.001 | <0.001 | -4 | -58 | 32 | Left Posterior Cingulate (BA 31) |
|  |  | 4.66 | 4.36 | <0.001 | <0.001 | -6 | -46 | 32 | Left Posterior Cingulate (BA 23) |
|  |  | 4.6 | 4.31 | <0.001 | <0.001 | -8 | -64 | 34 | Left Precuneus (BA 31) |
|  |  | 4.31 | 4.07 | <0.001 | <0.001 | -30 | -66 | 48 | Left Precuneus (BA 7) |
|  |  | 5.2 | 4.8 | <0.001 | <0.001 | -14 | -74 | 40 | Left Cuneus (BA 7) |
|  |  | 4.33 | 4.09 | <0.001 | <0.001 | 48 | -60 | 34 | Right Middle Temporal Gyrus (BA 39) |
|  |  | 4.87 | 4.53 | <0.001 | <0.001 | 50 | -70 | 16 |  |
|  |  | 4.73 | 4.41 | <0.001 | <0.001 | 42 | -78 | -10 | Right Fusiform Gyrus (BA 19) |
|  |  | 4.94 | 4.59 | <0.001 | <0.001 | 4 | -54 | 34 | Right Cingulate Gyrus (BA 31) |
|  |  | 5.13 | 4.74 | <0.001 | <0.001 | 4 | -46 | 24 | Right Posterior Cingulate (BA 30) |
|  |  | 5.06 | 4.68 | <0.001 | <0.001 | 4 | -44 | 28 | Right Posterior Cingulate (BA 23) |
|  |  | 4.61 | 4.32 | <0.001 | <0.001 | 8 | -52 | 28 | Right Posterior Cingulate (BA 31) |
|  |  | 4.45 | 4.19 | <0.001 | <0.001 | 60 | -48 | 24 | Right Supramarginal Gyrus (BA 40) |
|  |  | 4.29 | 4.05 | <0.001 | <0.001 | 62 | -44 | 44 |  |
|  |  | 4.44 | 4.18 | <0.001 | <0.001 | 40 | -56 | 54 | Right Inferior Parietal Lobule (BA 7) |
|  |  | 4.22 | 3.99 | <0.001 | <0.001 | 46 | -54 | 48 | Right Inferior Parietal Lobule (BA 40) |
|  |  | 4.21 | 3.98 | <0.001 | <0.001 | 42 | -72 | 34 | Right Superior Occipital Gyrus (BA 19) |
|  |  | 4.6 | 4.31 | <0.001 | <0.001 | 44 | -80 | -4 | Right Middle Occipital Gyrus (BA 18) |
|  |  | 4.35 | 4.1 | <0.001 | <0.001 | 24 | -50 | 64 | Right Precuneus (BA 7) |
|  |  | 4.2 | 3.98 | <0.001 | <0.001 | 32 | -46 | 62 |  |
|  |  | 5.15 | 4.76 | <0.001 | <0.001 | 22 | -68 | 40 |  |
|  |  | 4.79 | 4.47 | <0.001 | <0.001 | 28 | -50 | 48 |  |
|  |  | 4.15 | 3.93 | <0.001 | <0.001 | 6 | -70 | 36 | Right Precuneus (BA 31) |
|  |  | 4.38 | 4.12 | <0.001 | <0.001 | 28 | -76 | 38 |  |
|  |  | 4.54 | 4.26 | <0.001 | <0.001 | 62 | -30 | 26 | Right Insula (BA 13) |
|  |  | 4.32 | 4.08 | <0.001 | <0.001 | 36 | -66 | -18 | Right Cerebellum Posterior Lobe |
|  |  | 4.27 | 4.03 | <0.001 | <0.001 | 52 | -42 | 34 | No Grey Matter Found |
| <0.001 | 4893 | 5.26 | 4.84 | <0.001 | <0.001 | 20 | 46 | 32 | Right Superior Frontal Gyrus (BA 9) |
|  |  | 4.95 | 4.6 | <0.001 | <0.001 | 28 | 32 | 48 | Right Superior Frontal Gyrus (BA 8) |
|  |  | 3.5 | 3.36 | <0.001 | <0.001 | 16 | 16 | 56 | Right Superior Frontal Gyrus (BA 6) |
|  |  | 3.4 | 3.27 | <0.001 | 0.001 | 24 | 52 | 16 |  |
|  |  | 3.38 | 3.25 | <0.001 | 0.001 | 10 | 12 | 60 |  |
|  |  | 3.29 | 3.17 | <0.001 | 0.001 | 14 | 20 | 60 |  |
|  |  | 5.15 | 4.76 | <0.001 | <0.001 | 26 | 34 | 38 | Right Middle Frontal Gyrus (BA 8) |
|  |  | 4.89 | 4.55 | <0.001 | <0.001 | 30 | 28 | 50 |  |
|  |  | 4.21 | 3.98 | <0.001 | <0.001 | 32 | 20 | 56 |  |
|  |  | 3.72 | 3.56 | <0.001 | <0.001 | 32 | 18 | 44 |  |
|  |  | 3.99 | 3.8 | <0.001 | <0.001 | 38 | 30 | 40 | Right Middle Frontal Gyrus (BA 9) |
|  |  | 4.38 | 4.13 | <0.001 | <0.001 | 40 | 36 | 30 |  |
|  |  | 4.13 | 3.91 | <0.001 | <0.001 | 28 | 4 | 52 | Right Middle Frontal Gyrus (BA 6) |
|  |  | 3.51 | 3.37 | <0.001 | <0.001 | 42 | 0 | 54 |  |
|  |  | 3.01 | 2.91 | <0.001 | 0.002 | 44 | 46 | 14 | Right Middle Frontal Gyrus (BA 10) |
|  |  | 4.32 | 4.07 | <0.001 | <0.001 | 14 | 40 | 44 | Right Medial Frontal Gyrus (BA 8) |
|  |  | 4.1 | 3.89 | <0.001 | <0.001 | 12 | 34 | 48 |  |
|  |  | 4.1 | 3.88 | <0.001 | <0.001 | 10 | 50 | 40 |  |
|  |  | 3.38 | 3.25 | <0.001 | 0.001 | 28 | 52 | 8 | Right Medial Frontal Gyrus (BA 10) |
|  |  | 3.34 | 3.21 | <0.001 | 0.001 | 10 | 62 | 14 |  |
|  |  | 3 | 2.91 | <0.001 | 0.002 | 16 | 64 | 6 |  |
|  |  | 2.99 | 2.9 | <0.001 | 0.002 | 12 | 2 | 62 | Right Medial Frontal Gyrus (BA 6) |
|  |  | 3.94 | 3.75 | <0.001 | <0.001 | 46 | 6 | 36 | Right Precentral Gyrus (BA 6) |
|  |  | 3.89 | 3.71 | <0.001 | <0.001 | 34 | -8 | 58 |  |
|  |  | 3.52 | 3.38 | <0.001 | <0.001 | 56 | 4 | 36 |  |
|  |  | 3.39 | 3.26 | <0.001 | 0.001 | 38 | 10 | 30 |  |
|  |  | 3.74 | 3.57 | <0.001 | <0.001 | 40 | 14 | 42 | Right Precentral Gyrus (BA 9) |
|  |  | 2.95 | 2.86 | <0.001 | 0.002 | 56 | 8 | 8 | Right Precentral Gyrus (BA 44) |
|  |  | 3.33 | 3.21 | <0.001 | 0.001 | 8 | 20 | 44 | Right Cingulate Gyrus (BA 32) |
|  |  | 3.25 | 3.14 | <0.001 | 0.001 | 22 | 12 | 50 | Right Cingulate Gyrus (BA 24) |
|  |  | 3.16 | 3.06 | <0.001 | 0.001 | 46 | 14 | 22 | Right Insula (BA 13) |
|  |  | 3.18 | 3.08 | <0.001 | 0.001 | 24 | -2 | 60 | Right Frontal Sub-gyral (BA 6) |
| <0.001 | 1702 | 5.04 | 4.67 | <0.001 | <0.001 | -38 | 6 | 54 | Left Middle Frontal Gyrus (BA 6) |
|  |  | 4.92 | 4.57 | <0.001 | <0.001 | -22 | 28 | 44 | Left Middle Frontal Gyrus (BA 8) |
|  |  | 3.97 | 3.77 | <0.001 | <0.001 | -20 | 38 | 46 | Left Middle Frontal Gyrus (BA 9) |
|  |  | 3.83 | 3.65 | <0.001 | <0.001 | -36 | 42 | 28 |  |
|  |  | 3.42 | 3.29 | <0.001 | <0.001 | -30 | 40 | 38 |  |
|  |  | 2.84 | 2.76 | <0.001 | 0.003 | -42 | 20 | 34 |  |
|  |  | 4.78 | 4.46 | <0.001 | <0.001 | -24 | 16 | 50 | Left Medial Frontal Gyrus (BA 32) |
|  |  | 3.7 | 3.54 | <0.001 | <0.001 | -10 | 46 | 40 | Left Medial Frontal Gyrus (BA 8) |
|  |  | 3.03 | 2.93 | <0.001 | 0.002 | -8 | 30 | 48 |  |
|  |  | 4.36 | 4.11 | <0.001 | <0.001 | -14 | 16 | 62 | Left Superior Frontal Gyrus (BA 6) |
|  |  | 3.08 | 2.98 | <0.001 | 0.001 | -38 | 24 | 42 | Left Precentral Gyrus (BA 9) |
|  |  | 2.95 | 2.86 | <0.001 | 0.002 | -44 | 2 | 40 |  |
|  |  | 4.14 | 3.93 | <0.001 | <0.001 | -14 | 54 | 28 | Left Superior Frontal Gyrus (BA 9) |
|  |  | 3.24 | 3.13 | <0.001 | 0.001 | -10 | 22 | 50 | Left Cingulate Gyrus (BA 32) |
| 0.008 | 978 | 4.5 | 4.22 | <0.001 | <0.001 | 48 | 10 | -38 | Right Middle Temporal Gyrus (BA 21) |
|  |  | 4.4 | 4.15 | <0.001 | <0.001 | 64 | -12 | -20 |  |
|  |  | 3.61 | 3.46 | <0.001 | <0.001 | 58 | -6 | -34 | Right Inferior Temporal Gyrus (BA 20) |
| <0.001 | 1890 | 4.33 | 4.09 | <0.001 | <0.001 | -60 | -52 | -4 | Left Middle Temporal Gyrus (BA 37) |
|  |  | 3.6 | 3.45 | <0.001 | <0.001 | -62 | -38 | -14 | Left Middle Temporal Gyrus (BA 20) |
|  |  | 3.78 | 3.61 | <0.001 | <0.001 | -58 | -60 | 2 | Left Inferior Temporal Gyrus (BA 19) |
|  |  | 3.71 | 3.55 | <0.001 | <0.001 | -42 | -84 | -8 | Left Inferior Occipital Gyrus (BA 18) |
|  |  | 3.58 | 3.43 | <0.001 | <0.001 | -54 | -64 | -10 | Left Fusiform Gyrus (BA 19) |
|  |  | 3.4 | 3.27 | <0.001 | 0.001 | -58 | -56 | -14 | Left Fusiform Gyrus (BA 37) |
|  |  | 3.26 | 3.15 | <0.001 | 0.001 | -30 | -90 | -14 | Left Fusiform Gyrus (BA 18) |
|  |  | 3.98 | 3.78 | <0.001 | <0.001 | -22 | -64 | -10 | Left Cerebellum Posterior Lobe |
|  |  | 3.87 | 3.69 | <0.001 | <0.001 | -36 | -80 | -16 |  |
|  |  | 3.54 | 3.4 | <0.001 | <0.001 | -26 | -50 | -10 | Left Cerebellum Anterior Lobe |
|  |  | 3.24 | 3.12 | <0.001 | 0.001 | -26 | -22 | -30 |  |
|  |  | 2.88 | 2.8 | <0.001 | 0.003 | -26 | -32 | -24 |  |
|  |  | 2.75 | 2.67 | <0.001 | 0.004 | -22 | -36 | -18 |  |
|  |  | 2.67 | 2.6 | <0.001 | 0.005 | -16 | -42 | -12 |  |

Supplementary Table 4

| FWE p | Cluster Size (k) | T-value | Z-value | Cluster p (unc.) | Voxel p (unc.) | MNI Coordinates | | | Nearest Gray Matter Region |
| --- | --- | --- | --- | --- | --- | --- | --- | --- | --- |
|  |  |  |  |  |  | X | Y | Z |  |
| <0.001 | 8947 | 4.76 | 4.09 | <0.001 | <0.001 | -22 | 30 | -20 | Left Middle Frontal Gyrus (BA 11) |
|  |  | 4.32 | 3.79 | <0.001 | <0.001 | -44 | 28 | 30 | Left Middle Frontal Gyrus (BA 9) |
|  |  | 3.82 | 3.43 | <0.001 | <0.001 | -36 | 34 | 38 |  |
|  |  | 3.77 | 3.4 | <0.001 | <0.001 | -38 | 28 | 44 | Left Middle Frontal Gyrus (BA 8) |
|  |  | 3.91 | 3.5 | <0.001 | <0.001 | 0 | 30 | -22 | Left Medial Frontal Gyrus (BA 25) |
|  |  | 3.86 | 3.46 | <0.001 | <0.001 | -12 | 56 | 2 | Left Medial Frontal Gyrus (BA 10) |
|  |  | 4.07 | 3.61 | <0.001 | <0.001 | -46 | 20 | 14 | Left Inferior Frontal Gyrus (BA 44) |
|  |  | 3.88 | 3.48 | <0.001 | <0.001 | -40 | 40 | -4 | Left Inferior Frontal Gyrus (BA 47) |
|  |  | 3.77 | 3.39 | <0.001 | <0.001 | -34 | 38 | -8 |  |
|  |  | 3.74 | 3.37 | <0.001 | <0.001 | -6 | 28 | -14 | Left Subcallosal Gyrus (BA 25) |
|  |  | 3.83 | 3.44 | <0.001 | <0.001 | -10 | 42 | 10 | Left Anterior Cingulate (BA 32) |
|  |  | 3.83 | 3.44 | <0.001 | <0.001 | 42 | 28 | 32 | Right Middle Frontal Gyrus (BA 9) |
|  |  | 3.96 | 3.53 | <0.001 | <0.001 | 44 | 44 | 8 | Right Middle Frontal Gyrus (BA 10) |
|  |  | 4.83 | 4.14 | <0.001 | <0.001 | 24 | 30 | -20 | Right Middle Frontal Gyrus (BA 11) |
|  |  | 4.01 | 3.57 | <0.001 | <0.001 | 30 | 38 | -18 |  |
|  |  | 4.08 | 3.62 | <0.001 | <0.001 | 48 | 30 | 10 | Right Inferior Frontal Gyrus (BA 13) |
|  |  | 4.88 | 4.17 | <0.001 | <0.001 | 50 | 34 | 2 |  |
|  |  | 4.86 | 4.16 | <0.001 | <0.001 | 56 | -2 | -22 | Right Middle Temporal Gyrus (BA 21) |
|  |  | 4.74 | 4.08 | <0.001 | <0.001 | 40 | 6 | -34 |  |
|  |  | 4.67 | 4.03 | <0.001 | <0.001 | 44 | -6 | -30 |  |
|  |  | 4.04 | 3.59 | <0.001 | <0.001 | 50 | -20 | -28 | Right Inferior Temporal Gyrus (BA 20) |
|  |  | 4.42 | 3.86 | <0.001 | <0.001 | 40 | 12 | 40 | Right Precentral Gyrus (BA 9) |
|  |  | 4.37 | 3.82 | <0.001 | <0.001 | 36 | 10 | 36 | Right Precentral Gyrus (BA 6) |
|  |  | 3.94 | 3.52 | <0.001 | <0.001 | 6 | 24 | -16 | Right Subcallosal Gyrus (BA 25) |
|  |  | 3.75 | 3.38 | <0.001 | <0.001 | 8 | 36 | 8 | Right Anterior Cingulate (BA 24) |
|  |  | 3.89 | 3.48 | <0.001 | <0.001 | 30 | 4 | -34 | Right Uncus (BA 36) |
|  |  | 3.75 | 3.38 | <0.001 | <0.001 | 52 | 10 | 16 | Right Insula (BA 13) |
|  |  | 4.35 | 3.81 | <0.001 | <0.001 | 28 | 12 | -12 | Right Putamen |
|  |  | 4.08 | 3.62 | <0.001 | <0.001 | 24 | 18 | -10 |  |
|  |  | 3.74 | 3.37 | <0.001 | <0.001 | 36 | -8 | -6 |  |
|  |  | 4.19 | 3.7 | <0.001 | <0.001 | 26 | -22 | -24 | Right Cerebellum Anterior Lobe |

Supplementary Table 5

| FWE p | Cluster Size (k) | T-value | Z-value | Cluster p (unc.) | Voxel p (unc.) | MNI Coordinates | |  | Nearest Gray Matter Region |
| --- | --- | --- | --- | --- | --- | --- | --- | --- | --- |
|  |  |  |  |  |  | X | Y | Z |  |
| <0.001 | 2106 | 5.92 | 4.81 | <0.001 | <0.001 | -22 | 36 | 46 | Left Middle Frontal Gyrus (BA 8) |
|  |  | 4.18 | 3.69 | <0.001 | <0.001 | -36 | 28 | 44 |  |
|  |  | 4.7 | 4.05 | <0.001 | <0.001 | -36 | 20 | 34 | Left Middle Frontal Gyrus (BA 9) |
|  |  | 3.64 | 3.29 | <0.001 | <0.001 | -36 | 44 | 28 |  |
|  |  | 4.15 | 3.67 | <0.001 | <0.001 | -40 | 4 | 52 | Left Middle Frontal Gyrus (BA 6) |
|  |  | 4.47 | 3.9 | <0.001 | <0.001 | -20 | 10 | 56 | Left Medial Frontal Gyrus (BA 6) |
|  |  | 3.3 | 3.03 | <0.001 | 0.001 | -12 | 44 | 40 | Left Medial Frontal Gyrus (BA 8) |
|  |  | 4.27 | 3.76 | <0.001 | <0.001 | -22 | 18 | 48 | Left Superior Frontal Gyrus (BA 8) |
|  |  | 3.92 | 3.5 | <0.001 | <0.001 | -6 | 36 | 48 |  |
|  |  | 3.9 | 3.49 | <0.001 | <0.001 | -14 | 16 | 60 | Left Superior Frontal Gyrus (BA 6) |
|  |  | 3.55 | 3.22 | <0.001 | 0.001 | -16 | 52 | 30 | Left Superior Frontal Gyrus (BA 9) |
|  |  | 3.52 | 3.2 | <0.001 | 0.001 | -18 | 52 | 24 |  |
|  |  | 3.53 | 3.21 | <0.001 | 0.001 | -42 | 10 | 34 | Left Inferior Frontal Gyrus (BA 9) |
|  |  | 3.8 | 3.42 | <0.001 | <0.001 | -26 | 6 | 60 | Left Frontal Sub-Gyral (BA 6) |
|  |  | 3.59 | 3.26 | <0.001 | 0.001 | -20 | 8 | 62 |  |
|  |  | 3.8 | 3.42 | <0.001 | <0.001 | -44 | -2 | 48 | Left Precentral Gyrus (BA 6) |
|  |  | 3.46 | 3.16 | <0.001 | 0.001 | -36 | 4 | 30 |  |
|  |  | 3.39 | 3.11 | <0.001 | 0.001 | -38 | 8 | 32 |  |
|  |  | 3.1 | 2.87 | <0.001 | 0.002 | -46 | 0 | 40 |  |
|  |  | 3.09 | 2.86 | <0.001 | 0.002 | -50 | 4 | 36 |  |
|  |  | 3.23 | 2.97 | <0.001 | 0.001 | -8 | 26 | 44 | Left Cingulate Gyrus (BA 32) |
| <0.001 | 11032 | 4.22 | 3.72 | <0.001 | <0.001 | -42 | -70 | 32 | Left Middle Temporal Gyrus (BA 39) |
|  |  | 3.94 | 3.52 | <0.001 | <0.001 | -48 | -66 | 30 |  |
|  |  | 3.86 | 3.46 | <0.001 | <0.001 | -36 | -60 | 38 |  |
|  |  | 4.88 | 4.17 | <0.001 | <0.001 | -48 | -58 | 38 | Left Superior Temporal Gyrus (BA 39) |
|  |  | 3.94 | 3.52 | <0.001 | <0.001 | -4 | -22 | 28 | Left Cingulate Gyrus (BA 23) |
|  |  | 4.25 | 3.75 | <0.001 | <0.001 | -6 | -54 | 18 | Left Posterior Cingulate (BA 29) |
|  |  | 4.22 | 3.72 | <0.001 | <0.001 | -8 | -46 | 32 | Left Posterior Cingulate (BA 23) |
|  |  | 4 | 3.57 | <0.001 | <0.001 | -14 | -60 | 20 | Left Posterior Cingulate (BA 30) |
|  |  | 5.12 | 4.32 | <0.001 | <0.001 | -4 | -60 | 32 | Left Precuneus (BA 31) |
|  |  | 3.83 | 3.44 | <0.001 | <0.001 | -20 | -70 | 30 |  |
|  |  | 4.95 | 4.22 | <0.001 | <0.001 | 14 | -62 | 62 | Left Precuneus (BA 7) |
|  |  | 4.25 | 3.74 | <0.001 | <0.001 | -22 | -60 | 58 |  |
|  |  | 5.58 | 4.61 | <0.001 | <0.001 | -12 | -70 | 42 | Left Cuneus (BA 7) |
|  |  | 4.82 | 4.13 | <0.001 | <0.001 | -12 | -78 | 38 | Left Cuneus (BA 19) |
|  |  | 4.59 | 3.98 | <0.001 | <0.001 | 50 | -68 | 16 | Right Middle Temporal Gyrus (BA 39) |
|  |  | 4.03 | 3.58 | <0.001 | <0.001 | 50 | -60 | 36 |  |
|  |  | 4.41 | 3.86 | <0.001 | <0.001 | 58 | -48 | 28 | Right Supramarginal Gyrus (BA 40) |
|  |  | 3.94 | 3.52 | <0.001 | <0.001 | 58 | -48 | 38 |  |
|  |  | 4.02 | 3.58 | <0.001 | <0.001 | 56 | -22 | 40 | Right Postcentral Gyrus (BA 2) |
|  |  | 4.01 | 3.57 | <0.001 | <0.001 | 46 | -26 | 58 | Right Postcentral Gyrus (BA 3) |
|  |  | 3.97 | 3.54 | <0.001 | <0.001 | 50 | -20 | 48 |  |
|  |  | 4.31 | 3.78 | <0.001 | <0.001 | 6 | -54 | 36 | Right Cingulate Gyrus (BA 31) |
|  |  | 4.87 | 4.16 | <0.001 | <0.001 | 4 | -58 | 22 | Right Posterior Cingulate (BA 23) |
|  |  | 4.37 | 3.82 | <0.001 | <0.001 | 6 | -46 | 22 | Right Posterior Cingulate (BA 30) |
|  |  | 3.89 | 3.48 | <0.001 | <0.001 | 8 | -26 | 28 |  |
|  |  | 4.02 | 3.58 | <0.001 | <0.001 | 12 | -50 | 32 | Right Posterior Cingulate (BA 31) |
|  |  | 4.85 | 4.15 | <0.001 | <0.001 | 16 | -64 | 36 | Right Precuneus (BA 31) |
|  |  | 4.76 | 4.09 | <0.001 | <0.001 | 34 | -46 | 62 | Right Precuneus (BA 7) |
|  |  | 4.56 | 3.96 | <0.001 | <0.001 | 22 | -52 | 66 |  |
|  |  | 4.04 | 3.59 | <0.001 | <0.001 | 28 | -50 | 48 |  |
|  |  | 3.98 | 3.55 | <0.001 | <0.001 | 60 | -28 | 22 | Right Insula (BA 13) |
|  |  | 4.42 | 3.86 | <0.001 | <0.001 | 40 | -78 | -12 | Right Cerebellum Posterior Lobe |
| <0.001 | 3884 | 5.48 | 4.55 | <0.001 | <0.001 | 34 | 22 | 54 | Right Middle Frontal Gyrus (BA 8) |
|  |  | 4.52 | 3.93 | <0.001 | <0.001 | 26 | 34 | 48 |  |
|  |  | 4.78 | 4.1 | <0.001 | <0.001 | 42 | 24 | 36 | Right Middle Frontal Gyrus (BA 9) |
|  |  | 4.61 | 3.99 | <0.001 | <0.001 | 40 | 20 | 38 |  |
|  |  | 3.49 | 3.18 | <0.001 | 0.001 | 40 | 38 | 28 |  |
|  |  | 3.17 | 2.93 | <0.001 | 0.002 | 34 | 38 | 32 |  |
|  |  | 4.35 | 3.81 | <0.001 | <0.001 | 42 | 0 | 52 | Right Middle Frontal Gyrus (BA 6) |
|  |  | 3.75 | 3.38 | <0.001 | <0.001 | 36 | 8 | 46 |  |
|  |  | 3.14 | 2.91 | <0.001 | 0.002 | 26 | 2 | 52 |  |
|  |  | 3.12 | 2.89 | <0.001 | 0.002 | 34 | -4 | 48 |  |
|  |  | 3.33 | 3.05 | <0.001 | 0.001 | 48 | 18 | 22 | Right Middle Frontal Gyrus (BA 46) |
|  |  | 4.84 | 4.15 | <0.001 | <0.001 | 20 | 36 | 46 | Right Medial Frontal Gyrus (BA 8) |
|  |  | 4.18 | 3.69 | <0.001 | <0.001 | 8 | 48 | 42 |  |
|  |  | 3.31 | 3.04 | <0.001 | 0.001 | 8 | 30 | 52 |  |
|  |  | 4.68 | 4.04 | <0.001 | <0.001 | 24 | 34 | 38 | Right Medial Frontal Gyrus (BA 6) |
|  |  | 3.22 | 2.97 | <0.001 | 0.001 | 14 | 16 | 54 | Right Medial Frontal Gyrus (BA 32) |
|  |  | 4.8 | 4.12 | <0.001 | <0.001 | 20 | 46 | 34 | Right Superior Frontal Gyrus (BA 9) |
|  |  | 3.12 | 2.88 | <0.001 | 0.002 | 12 | 56 | 30 |  |
|  |  | 4.63 | 4 | <0.001 | <0.001 | 24 | 26 | 50 | Right Superior Frontal Gyrus (BA 8) |
|  |  | 3.72 | 3.36 | <0.001 | <0.001 | 14 | 22 | 62 | Right Superior Frontal Gyrus (BA 6) |
|  |  | 4.68 | 4.04 | <0.001 | <0.001 | 52 | 6 | 30 | Right Inferior Frontal Gyrus (BA 9) |
|  |  | 4.5 | 3.92 | <0.001 | <0.001 | 28 | 0 | 66 | Right Frontal Sub-Gyral (BA 6) |
|  |  | 4.26 | 3.75 | <0.001 | <0.001 | 36 | -6 | 62 | Right Precentral Gyrus (BA 6) |
|  |  | 3.94 | 3.52 | <0.001 | <0.001 | 34 | -12 | 68 |  |
|  |  | 2.78 | 2.61 | <0.001 | 0.005 | 42 | -14 | 64 | Right Precentral Gyrus (BA 4) |
|  |  | 2.78 | 2.61 | <0.001 | 0.005 | 44 | -18 | 64 |  |
|  |  | 4.78 | 4.1 | <0.001 | <0.001 | 44 | 20 | 42 | Right Precentral Gyrus (BA 9) |
|  |  | 3.56 | 3.24 | <0.001 | 0.001 | 26 | 12 | 48 | Right Cingulate Gyrus (BA 32) |
|  |  | 2.84 | 2.66 | <0.001 | 0.004 | 54 | 12 | 20 | Right Insula (BA 13) |
